# Multi-level factors associated with utilization of water, sanitation and hygiene services by mothers in Nepal

**DOI:** 10.1371/journal.pone.0283379

**Published:** 2024-03-20

**Authors:** Shalik Ram Dhital, Catherine Chojenta, Deborah Loxton

**Affiliations:** 1 Ministry of Health and Population, National Health Education, Information and Communication Center, Kathmandu, Nepal; 2 Centre for Women’s Health Research, College of Health, Medicine and Wellbeing, University of Newcastle, Callaghan, New South Wales, Australia; 3 School of Health and Allied Sciences, Pokhara University, Lekhnath, Nepal; 4 Concern Center for Rural Youth, Kathmandu, Nepal; Gadjah Mada University Faculty of Medicine, Public Health, and Nursing: Universitas Gadjah Mada Fakultas Kedokteran Kesehatan Masyarakat dan Keperawatan, INDONESIA

## Abstract

**Background:**

Providing improved water, sanitation, and hygiene (WASH) at a household level remains one of the major public health challenges in Nepal. Household mothers are likely to have limited access to combined WASH services, this is influenced by individual, and community factors. Individual components of an improved water source, sanitary toilet, fixed place for handwashing, and availability of soap and water were merged into one and called combined WASH. This paper aimed to identify the individual and community factors associated with combined WASH facilities and practices among mothers with children under five years in Nepal.

**Methods:**

A cross-sectional study was conducted using data from the Nepal Demographic and Health Survey (NDHS), 2016. The weighted sample size of this study was 4887 mothers with children under five years. The independent variables within the mothers included age, education, occupation, and caste/ethnicity in addition to education of the husband, wealth index, exposure to the newspaper, radio and television, residence, ecological zones, provinces, distance and participation in health mother groups were analyzed with the outcome variable of combined WASH. A multi-level mixed effects logistic regression model was used to assess the relationship of explanatory variables with WASH.

**Results:**

At an individual level, a rich wealth index was positively associated with combined WASH (AOR = 6.29; 95%CI: 4.63–8.54). Higher education levels and exposure to television had higher odds of having combined WASH. At the community level, the hill zone, urban residence, and Sudurpashim Provinces were positively associated with combined WASH while Madesh and Karnali Provinces and distance to water source greater than 31 minutes were associated with lower access to combined WASH.

**Conclusion:**

Educated and rich household have positive association with combined WASH. It is recommended that both the health and other sectors may be instrumental in improving the combined WASH service for mothers at households.

## Introduction

In 2021, approximately 96% of Nepalese households had improved water sources, 84% had access to a sanitary toilet, 81% had a fixed place for handwashing with soap, and 47% had soap and water available in a handwashing place [1, 2]. There are few studies on individual household water, sanitation and hygiene (WASH) practices, and these do not specifically investigate the risk factors associated with mothers’ access to combined WASH in Nepal. The combined WASH covered an improved water source, a sanitary toilet, a fixed place for handwashing, and the availability of soap and water in the handwashing places. Mothers experience low-priority access to WASH facilities in their household due to demographic, geographical, economic and socio-cultural factors [3]. Mothers from Nepal, primarily undertake work as housewives and caretakers of their children and family members [4]. Mothers are primary carriers of water from the water source to their home [5]. Research has shown mothers in Nepal repeatedly clean and dispose of children’s faecal matter without protective equipment [6]. However, they are not allowed to use and carry water when they are menstruating [7]. The long distance between the home and water source can lead to psychological and emotional stress [8], which can subsequently impact mostly the mothers.

Mothers are the first teachers of their children, and based on scientific findings [9, 10], children are expected to adopt their mothers’ WASH practices. Mothers in Nepal can be informed of current WASH practices through community health mother group discussions, female community health volunteers, local teachers, health workers, and through mass media. When mothers meet and share their practices this can help develop good hygiene and sanitation habits amongst themselves and therefore amongst their children at home, which can prevent the spread of communicable diseases such as diarrhoea among children under five years [11, 12]. In Nepal, mothers have a primary responsibility to perform housekeeping tasks, such as ensuring an adequate water supply in the home, keeping the toilet clean, and the establishment of a handwashing place with sufficient soap and water [7]. Evidence has shown that mothers’ active participation in the planning and design of WASH interventions at the household level is more effective than the fathers’ [5, 13]. A mother’s leading role in the establishment of WASH in the household, increased the sustainability of good WASH practices [14].

The identification of the key risk factors for poor WASH uptake among mothers is important in developing strategies for improving WASH uptake. Previous assumptions relating to poor uptake of quality WASH practices have been linked to mothers who are older, uneducated, single, scheduled caste and Muslim, who bore children at an early age and breastfeed their children, work in agriculture, have minimal exposure to media, and reside in rural areas [15]. Consistent with Bronfenbrenner’s Socio-Ecological Model [16, 17] posits the level of WASH uptake is subject to multiple risk factors. However, the influence of individual factors [7, 18–21] and community factors [22] on WASH access for mothers at the household level are yet to be confirmed. This knowledge gap of not covering such variables and rationale for giving combined WASH requires further exploration by studying the WASH status of the sub-population of mothers in Nepal. The aim of this paper was to identify the individual and community factors associated with combined water, sanitation and hygiene (WASH) facilities and practices among mothers with children under five years in Nepal.

## Methods

### Data source and respondents

This study uses the nationally representative and publicly available NDHS 2016 datasets. For the purpose of this study, variables obtained from the children’s recode (KR) file and derived from individually recoded mothers’ data were considered. Study variables were taken from interview respondents’ (mothers) basic information, and maternity and child health. A number of WASH variables were taken from the household data. The total 4887 mothers were included to analyze the data for this study. The datasets were merged into one, where primary or base file was individual mothers recode (IR) which matched with household recode (HR) file from many entities to one entity (m:1) aimed to merge necessity household characteristics.

### Inclusion and exclusion criteria

Study population include usual residents (De Jure residence) of the household, who were mothers, aged 15–49 years, and who had children under five years on the day of survey. Mothers whose children died between birth and 36 months of age were excluded from this study after weighting the sample. There were no deaths recorded for children aged two to five years. Guest (De Facto residence) mothers were excluded from this study to avoid duplication of participants. The final number of study population was 4887.

### Description of dependent/outcome variables

The outcomes included in this study were a combined WASH which covered an improved water source, a sanitary toilet, a fixed place for handwashing, and the availability of soap and water in the handwashing places were all required, and the absence of any single item resulted in incomplete WASH. If these four criteria were met, it can be considered ‘complete WASH’. The dependent variable was categorized by dichotomous outcome, Yes = 1, No = 0.

### Description of independent variables

The multilayered individual and community factors for each outcome variables were extracted from the 2016 NDHS datasets. The individual and community level factors considered in this study are based on the Socio-ecological model [16, 17]. Variables are grouped according to previous literature and existing knowledge. All variables were validated using principal components analysis.

The individual level factors were age, education, occupation, caste or ethnicity, religion of mothers, education of husband, wealth index, exposure to newspaper, exposure to radio, and exposure to television. Family level factors were considered as individual factors because individual mothers use various outsourced household services. The community level factors were place of residence, ecological zone, provinces, distance to water source, and access to a health mothers group.

The multi-level factors of individual and community were described, coded and measured as Table 1 below:

**Table 1:** Description of independent variables.

Independent variables	Description
**Ages of the mothers**	Ages of the mothers were calculated based on v012 (*the current age of the mothers in year*). The mothers’ ages were recorded from their histories given during the survey. The age groups were recoded and generated a new variable *(women_age*), in three categories. These category were 15–24 = 1, 25–34 = 2, and 35 above = 3.
**Education of mothers**	The education of mother (v106) was categorized into four groups which was labelled as No education (unable to read and write) = 0, Primary (Year Five) = 1, Secondary (Year Eight) = 2, and School Leaving Certificate (Year ten), and Higher = 3.
**Occupation of mothers**	Eligible interviewed mothers were categorized by their occupation (v717) as No work = 1, Professional and manager = 2, Office administration = 3, Sales/Service = 4, Agricultural = 5, Skilled manual = 8, and Unskilled manual = 9. Occupation was also recoded into three categories, which were No work = 1, Agriculture = 2, and Non-agriculture = 3, and the recoded variable was *occupation_women*.
**Caste/ethnicity of mothers**	Caste/ethnicity was originally categorized as Hill Brahmin = 1, Hill Chhetri = 2, Plain Brahmin/Chhetri = 3, Other terai caste = 4, Hill Dalit = 5, Plain Dalit = 6, Newar = 7, Hill Janajati = 8, Plain Janajati = 9, Muslim = 10, and Other = 96. Later, the caste or ethnicity was recoded as (*ethnicity_category*) and labelled as- Brahmin and Chhetri = 1, Janajati/Vaishya = 2, Scheduled or Shudra caste = 3, and other = 4.
**Religion of the mothers**	The original category of religion (v130) was Hindu = 1, Buddhist = 2, Muslim = 3, Kirat = 4 and Christian = 5. This variable was recoded (*religion*) and categorized into two groups Hindu = 1 and Non-Hindu = 2 for this study.
**Education of husband**	Education of husband (v701) was recoded and created new variable *hus_education* which was further categorized into four groups, No education (unable to read and write) = 0, Primary (Year Five) = 1, Secondary (Year Eight) = 2, and School Leaving Certificate (Year Ten) and Higher = 3.
**Wealth index**	The wealth index (v190) was labelled as Poorest = 1, Poorer = 2, Middle = 3, Richer = 4, and Richest = 5. The wealth index was further recoded as Poor = 1, Middle = 2, and Rich = 3, and created a new variable as *women_windex* and Poorer and Poor and Richer and Rich were collapsed.
**Exposure to newspaper**	Exposure to newspaper/magazine (v157) was categorized into three groups: Not at all = 0, Less than once a week = 1, and At least once a week = 2. These were collapsed into two categories, Non-exposure = 0 and Exposure = 1, because of the low sample. The new variable was *newspaper*.
**Exposure to radio**	Exposure to radio (v158) was categorized into three groups: Not at all = 0, Less than once a week = 1, and At least once a week = 2. These were collapsed into two categories as Non-exposure = 0 and Exposure = 1, because of the low sample. The new variable was *radio*.
**Exposure to television**	Exposure to television (v159) was categorized into three groups: Not at all = 0, Less than once a week = 1, and At least once a week = 2. These were collapsed into two categories as Non-exposure = 0 and Exposure = 1 because of the low sample. The new variable was TV.
**Place of residence**	The existing NDHS categories for place of residence (stype1) was categorized into two groups Rural = 1 and Urban = 2.
**Ecological zone**	The three ecological zones were categorized as Plain = 1, Hills = 2 and Mountain = 3, and the new variable was *geography_type*.
**Provinces**	The existing NDHS categories for provinces were retained, with no change. These are named as Koshi, Province, Madesh Province, Bagmati Province, Gandaki Province, Lumbini Province, Karnali Province, and Sudurpashchim Province; starting from eastern part of Nepal towards the west.
**Distance to water source**	This variable was modified from the NDHS term ‘time to get to water source’ and the discrete number of distribution was categorized into two groups, ≤30 minutes and >31 minutes. Water source within home premises and <30 minutes were merged due to a low sample and were considered as water source<30 minutes. These were categorized into two groups, ≤30 minutes = 1 and >31 minutes = 2. The new variable was named as *distance_water*.
**Health Mother Group**	Health Mother Group (HMG) is a non-political women’s organization in the community. Each HMG in the mountain region of Nepal has at least 11 people, 15 in the hilly region, and 21 in the plain region. The HMG (s1108bb) was categorized into three groups, HMG Not available = 0, HMG available = 1, and Don’t know = 8, with no change from NDHS category.

Above variables were categorized according to the classification of previously published articles [1, 23], PhD thesis [24] and DHS-7 guidelines [25].

### Data analysis

STATA 15 was used to analyze the study data [26]. The descriptive analysis was calculated using survey weights, and frequency and percentage were reported. All socio-demographic and contextual WASH related characteristics were analyzed through a univariate method. A weighted sample was considered for the analysis to match population distribution.

A multi-level mixed effects logistic regression analysis [27] was performed to assess the relationship of explanatory variables at individual and community levels [16]), with each binary outcomes of combined WASH among mothers with children under five years in Nepal. The NDHS employed a multistage cluster sampling technique where mothers were hierarchical (nested) within households, which were nested within regions/provinces. Due to the hierarchical nature of NDHS, a two level hierarchical generalized linear model (HGLM) was used for the purpose of this study. The traditional logistic regression model undertakes independency among observations. The data with a hierarchical nature however, often has a dependency within a higher level of hierarchy. The multi-level modelling was applied to take this effect into account [27, 28].

This analysis was performed using STATA 15 to estimate hierarchical linearized model. The equation applied for this model is shown below:

$$Y_{ij}=\gamma_{00}+\gamma_{01}+W_{j}+\mu_{0j}+\gamma_{10}X_{ij}+\mu_{1j}X_{ij}$$

In this model, Yij characterizes the log odds of using each WASH component facilities for mother *i* in region *j*, ***γ*00** provides the log odds of using WASH facilities in a typical region, *Wj* is a region level predictor for region *j*, ***γ*01** is the slope related with this predictor, *μ***0*j*** is the level 2 error term representing a unique effect associated with region *j***, *γ*10** is the average effect of the individual level predictor, *X****ij*** is an individual level predictor for mothers i in region *j*, and *μ***1*j*** is a random slope for a level-1 predictor variable *X****ij***, which allows the relationship between the individual level predictor (*X****ij***) and the outcome (*Y****ij***) to differ across level 2 units.

Three analysis were conducted on the dependent variables using NDHS 2016 dataset.

**Model 1:** All individual level variables were run together and the difference between unadjusted models was observed. This model was run to examine the contribution of each individual level factors on outcomes. If effect sizes changed by more than 10% between crude and adjusted models, this indicates some confounders may be occurring. Variance Inflation Factor (VIF) was checked to assess collinearity. The cutoff point value of VIF was less than 5 which is included in the model.

**Model 2:** All community level variables were run together and the difference between unadjusted models and individual level factors analysis was observed. If effect size changed by more than 10% between crude and adjusted models, this indicates some confounders may be occurring. Variance Inflation Factor (VIF) was checked to assess collinearity. The cutoff point value of VIF was less than 5 which is included in the model.

**Model 3:** Individual and community level variables were combined and the model for final evaluation was run. Each outcome of this study were carefully analyzed, adopting similar processes, as Model 1 and 2 above. VIF was calculated to assess collinearity. The cutoff point value of VIF was less than 5 which is included in the model. As illustrated in the S1 Fig in the supporting information, the Hosmer-Lemeshow goodness of fit (GOF) test was applied to assess the fitness of the model [29].

In this model, measure of relationship reported an Adjusted Odds Ratio (AOR) with 95% Confidence Interval (CI), by controlling the effects of other predictors. P-value of less than 0.05 in line with Odds ratio not in 1 was applied to identify factors significantly associated with combined WASH for mothers.

### Socioecological model

The socioecological model [17, 30] as a new public health (health promotion) approach focuses on individual, family, community and policy-level factors which indicated that these levels of factors are called micro, meso and macro level factors respectively. This paper included micro level factors of WASH with individual and family level components because individual mothers and household family members share similar access to combined WASH, as illustrated in S2 Fig in the supporting information. The meso level factors are defined as community level factors and macro level factors are considered as policy level factors where this level of factors were not included in this paper due to lack of data available.

### Ethics statement

Ethical approval for this study was obtained from the Human Research Ethics Committee of the University of Newcastle (Ref. No: H-2018-0511). Prior to analysis, we obtained permission from the Inner City Fund Institutional Review Board, the Demographic Health Survey Program. The Ethical Review Board of the Nepal Health Research Council, Kathmandu, Nepal Provided ethical approval prior to the NDHS in 2016. Respondents were informed about what was involved in participating in the survey and gave written consent to the interview. Respondents were assured their personal details would remain confidential.

## Results

### Descriptive statistics

Supporting Information (S1 Table) shows the distribution of socio-demographic and contextual WASH characteristics of the respondents. The age group with the highest representation in the study was 25–34 years, comprising 51.3% of the total respondents. Approximately 32% of respondents had a secondary level of education and 24% had no education. The majority of mothers, constituting 45.2%, engaged in agricultural work at home. Additionally, a significant portion, accounting for 40.8% were identified as not being employed. Approximately 38.2% of respondents identified as Janajati or Vaishya caste, while approximately 85.2% identified as being Hindu.

Of respondents’ husbands, 14.7% had no education, 44.5% were literate with secondary level of education, and half had a primary level of education. Almost 40% of husbands undertook manual work, while 2.2% had no work. Forty-two percent of respondents were classified as being in the poor wealth index category, with 35.4% classified as rich. The proportion of respondents in the middle wealth index was 22.3%. The proportion of respondents exposed to media at any time was 21.7% for newspapers, 50.3% for radio, and 61.5% for television. However, the proportion of respondents exposed to media at least once a week was 16.6%, 27.5%, and 20.5% for newspaper, radio and television, respectively.

Of respondents’ places of residence, 61.9% were in rural areas and 38.8% were in urban areas. With regard to respondents’ ecological zone location, 55% were in plain, 38% in hill, and 7% in mountain regions. Provincial representation of respondents was unequal, with the highest proportion at 26.8% being from Madesh Province and lowest at 6.6% from Karnali Province. Eighty-seven percent of respondents’ homes were less than 30 minutes walking distance from water source. Approximately 33% of respondents had a HMG available in the community. The overall combined WASH status for mothers of Nepal was 32.7%.

### Results of individual and community factors relating to combined WASH services

The age of the mother, the education of mother and her husband, caste and ethnicity, wealth index, and exposure to television are significantly associated with combined WASH for mothers at home (Table 2). When compared to very young mothers, being those of the 15–24 years’ age group, mothers aged 25–34 years were more likely to have combined WASH (AOR = 1.21; 95%CI: 1.00–1.46). Mothers who obtained secondary and SLC or higher level of education had higher odds (AOR = 1.48; 95%CI: 1.12–1.96 and AOR = 2.12; 95%CI: 1.48–3.08, respectively) of having combined WASH compared with mothers who had no education.

**Table 2:** Multi-level analysis of individual and community factors associated with combined WASH for mothers with children under five years.

Variables	Classes	Model 1 AOR(95% CI)	Model 2 AOR(95% CI)	Model 3 AOR(95% CI)	P- value
**Age of mother (years)**	15–24	1		1	
	25–34	**1.32(1.10–1.58)**		**1.21(1.00–1.46)**	**0.050**
	35 above	1.03(0.73–1.46)		0.89(0.62–1.29)	0.551
**Education of mother**	No education	1		1	
	Primary	1.15(0.88–1.49)		1.06(0.81–1.40)	0.663
	Secondary	**1.61(1.23–2.09)**		**1.48(1.12–1.96)**	**0.006**
	SLC or higher	**1.96(1.38–2.77)**		**2.12(1.46–3.08)**	**<0.001**
**Occupation of mother**	No work	1		1	
	Agriculture	**1.07(0.87–1.33)**		0.91(0.72–1.14)	0.407
	Non agriculture	**1.30(1.01–1.68)**		1.15(0.87–1.52)	0.327
**Caste/ethnicity**	Brahmin/Chhetri	1		1	
	Janajati/Vaishya	**1.33(1.04–1.70)**		**1.47(1.12–1.92)**	**0.006**
	Scheduled	1.14(0.84–1.54)		1.23(0.89–1.70)	0.203
	Others	**0.68(0.47–0.97)**		0.99(0.66–1.49)	0.954
**Religion**	Hindu	1		1	
	Non Hindu	0.90(0.66–1.22)		0.98(71–1.36)	0.909
**Education of husband **	No education	1		1	
	Primary	**1.66(1.18–2.32)**		**1.49(1.05–2.11)**	**0.025**
	Secondary	**1.96(1.42–2.71)**		**1.86(1.33–2.59)**	**<0.001**
	SLC or higher	**2.56(1.75–3.75)**		**3.01(2.00–4.50)**	**<0.001**
**Wealth index**	Poor	1		1	
	Middle	**2.14(1.66–2.76)**		**2.12(1.61–2.79)**	**<0.001**
	Rich	**6.12(4.66–8.03)**		**6.29(4.63–8.54)**	**<0.001**
**Exposure to newspaper**	Not exposure	1		1	
	Exposure	1.19(0.95–1.49)		1.14(0.90–1.46)	0.279
**Exposure to radio **	Not exposure	1		1	
	Exposure	0.96(0.79–1.16)		1.02(0.83–1.26)	0.838
**Exposure to television**	Not exposure	1		1	
	Exposure	1.62(1.31–2.00)		**1.75(1.41–2.18)**	**<0.001**
**Place of residence**	Rural		**1**	**1**	
	Urban		**4.37(3.11–6.13)**	**1.95(1.39–2.73)**	**<0.001**
**Ecological zone**	Plain		1	1	
	Hill		0.97(0.60–1.56)	**1.67(1.04–2.67)**	**0.033**
	Mountain		**0.40(0.19–0.83)**	0.86(0.42–1.74)	0.672
**Provinces**	Koshi		1	1	
	Madesh		**0.32(0.17–0.59)**	**0.51(0.27–0.94)**	**0.032**
	Bagmati		**2.04(1.07–3.87)**	1.25(0.67–2.33)	0.490
	Gandaki		1.23(0.65–2.35)	0.90(0.48–1.68)	0.733
	Lumbini		1.00(0.56–1.81)	1.01(0.57–1.78)	0.973
	Karnali		**0.36(0.18–0.70)**	**0.53(0.27–1.01)**	**0.055**
	Sudurpashchim		1.14(0.62–2.07)	**1.99(1.11–3.59)**	**0.022**
**Distance to water source**	≤30 mins		1	1	
	>31 mins		**0.26(0.17–0.41)**	**0.36(0.22–0.58)**	**<0.001**
**Health Mother Group**	No		1	1	
	Yes		1.17(0.95–1.43)	1.01(0.81–1.27)	0.903
	Don’t know		1.24(0.95–1.61)	1.11(0.83–1.48)	0.488

Mothers’ whose husband had no education were less likely to have access to combined WASH than mothers whose husband had at least a low level of education (Table 2). Mothers identifying as Janajati and Vaishya caste had higher odds (AOR = 1.47; 95%CI: 1.12–1.92) of having combined WASH compared to those identifying as Brahmin and Chhetri caste. Mothers of the rich wealth index had six fold higher odds of combined WASH (AOR = 6.29; 95%CI: 4.63–8.54) compared to those of the poor wealth index. Also, mothers of the middle wealth index had two fold higher odds of combined WASH (AOR = 2.12; 95%CI: 1.61–2.79) compared to those of the poor wealth index. Mothers who had access to television had higher odds (AOR = 1.75; 95%CI: 1.41–2.18) of having combined WASH than mothers without access to television.

Mothers’ places of residence, ecological zone, Province and distance to a water source were significantly associated with combined WASH. Mothers who lived in urban areas were 1.95 times more likely (AOR = 1.95; 95%CI: 1.39–2.73) to have combined WASH compared with mothers residing in rural areas. Mothers residing in hill regions had higher odds (AOR = 1.67; 95%CI: 1.04–2.67) of having combined WASH compared with mothers in plain regions. Mothers from Madesh Province had lower odds (AOR = 0.51; 95%CI: 0.27–0.94) and mothers from Sudurpashchim Province had higher odds (AOR = 1.99; 95%CI: 1.11–3.59) of having combined WASH compared with mothers from Koshi Province. Mothers who had to walk further than 31 minutes from home to reach a water source had lower odds (AOR = 0.36; 95%CI: 0.22–0.58) of having combined WASH, compared with mothers who lived 30 minutes or less from a water source.

## Discussion

This is the first known study involving the multi-level analysis of maternal combined WASH access in Nepal. This present study identified a number of factors which have a significant influence on mothers’ use of and access to an improved water source, a sanitary toilet, a fixed place for handwashing, and on availability of soap and water in the handwashing place, and also access to the combination of these components, termed ‘combined WASH’. The individual and community level factors were methodologically considered as a multi-level analysis [31]. A multi-level hierarchical regression model was appropriate in this study as the data were organized at more than one level (individual and community levels) [32]. Results showed ten individual and five community factors had a significant influence on combined WASH services for mothers with children under five years.

### Individual level factors and combined WASH

In this study, mother’s ages were found to be associated with having access to combined WASH service, a finding which is supported by a previous study conducted in Nepal where participants aged 30–40 years had a significant association with good sanitation [33].

Mothers and their husbands with at least a basic level of education had a greater understanding of the benefits of mothers having access to combined WASH, compared to mothers and husbands with no education. Relative to mothers with no education, mothers who were well educated (with secondary and higher education) had greater confidence in undertaking improved WASH, as increased knowledge of these practices encourages mothers to adhere to WASH standards. These findings are supported by evidence-based literature on developing countries, such as studies conducted in the South Asia Region [34, 35]. It is evident that education is crucial in improving WASH uptake at the household level, as educated mothers and family members are more aware of the risks associated with poor WASH practices.

In the current study, the occupation of the mother in the household was insignificantly associated with the outcome of combined WASH. By contrast, a previous study conducted in India showed mothers’ occupations were significantly associated with all WASH components [36]. The difference in these results may be due to sample size, sociodemographic status, and/or contextual factors. A qualitative study conducted in Indonesia among women in 2019 supports the theory that the economically sound women had good decision-making power and networking capability, which can facilitate better access to improved WASH [37].

Mothers of the Janajati or Vaishya castes had higher prevalence of having a fixed place for handwashing, and therefore greater access to combined WASH than mothers from other castes. WaterAid’s research raises issues regarding the Nepalese Government’s commitment to achieving universal access to sanitation and hygiene, particularly for mothers, ethnic groups, those of a lower caste, and disabled people in the country [38], which supports this present study’s recommendation.

Mothers also experience a lack of autonomy in household WASH practices because they have low priority for the use of these services in patriarchal rural communities of Nepal [38]. The present study is similar to a previous study conducted in Ghana in 2018 that showed households with the mother as its head demonstrated good WASH practices, when mothers are the head of the households they are more likely to be in the high wealth index [39]. Economic status determines wealth index, location of residence, and distance to a water source. Those of a lower wealth index have limited household WASH facilities, poor living standards, and reside a long distance from water [40]. Mothers’ exposure to television has a significant association with combined WASH. Mothers who have access to mass media experience further exposure to relevant information on hygiene and sanitation which enables them to be aware of and to seek adequate WASH services at home.

### Community level factors and combined WASH

Ecological zone, place of residence and distance to a water source were significantly associated with combined WASH outcomes. Mothers of poor socioeconomic status, with residences in rural remote and/or mountainous areas, and with long distances to water sources from home had poor access to WASH. The urban mothers were more likely be employed and were therefore of a higher wealth index than mothers in rural areas and were then more likely to have improved WASH services in the households. These mothers would have the necessary resources to build sanitary toilets, to install plumbing for water access inside the residence, and the capacity to purchase soap and other cleaning and sanitary items. Mothers from hill zones had good access and use of combined WASH.

The distribution of WASH components by Province was found to be varied, with the lowest levels of access to WASH experienced in Madesh Province in the plain region. This may be due to the low level of education, high poverty, gender discrimination, and low level of WASH provision in this Province [41]. Mothers in Sudurpashchim Province reported the highest use and access to WASH, which may be due to targeted WASH programs facilitated by government and non-government organizations, such as Rural Village Water Resources Management Project with technical support of the Finnish Government in this Province [7]. That project provides support similar to the Vietnam WASH services, where handwashing practice was highest in the world due to the commitment of the government of Vietnam, with the support of Danish Government [42].

The findings of this study, regarding the prevalence of improved water source, sanitary toilet, and handwashing with soap facilities are consistent with those of several previous studies [43–45]. People from rural areas and those who lived a longer distance from a water source, as well as those who lived in the plain regions, had poorer WASH access than those who lived in hill region and urban areas. This present study suggests that community level factors have greater impact on mothers’ WASH than individual level factors. Educating mothers on the health benefits of a combined WASH is imperative. In the household, the mother is responsible for child care and grooming and for the housework and maintenance, therefore the quality of family life is influenced greatly by her management of daily activity. Mothers in Madesh Province had lower rates of combined WASH services while those in Sudurpashchim Province had higher rates.

These findings have identified the need for, and value of, context-specific policy, and effective strategies for the promotion of WASH practices from a maternal perspective. Further research is required to determine the relationship between individual WASH components as well as combined WASH and communicable disease in Nepal. Additionally, geopolitical-based surveys could be conducted in relation to the political structure of Nepal, including schools, colleges, health facilities, and public and private organizations, in line with Sustainable Development Goals (2016–30) targets related to WASH and beyond. This could provide a more comprehensive understanding of WASH for mothers, as well as the broader population. Additionally, an evaluation of the impact of WASH practices and facilities on Nepalese mothers’ children under five years and their health could be conducted. Lastly, updating policies and strategies focusing on combined WASH it is a recommended aim to influence a change in working mindsets through the global best practices consistent with the Ottawa Charter health promotion mandate to meet Sustainable Development Goals (2016–30) [46].

### Strengths and limitations

This study used NDHS 2016 data which had a large sample size as data were collected from 383 clusters from 14 rural and urban strata. Mothers were selected from the systematic randomly selected households, reducing selection bias [47].

The study applied multi-level modelling to complete the hierarchical nature of the dataset. Multi-level modelling was more appropriate than classical logistic regression which helps with data reduction [47]. This study focused on mothers, as their WASH practices have a significant influence on the health of their children [48]. Consideration and analysis of sub-population level factors could inform policy makers, including political leaders and public health organizations, of the importance of WASH for mothers of children under five years. When mothers have improved access WASH services, other household members are more likely to also use these services. Mothers have the potential to be the agents for change in this area and to establish credibility of WASH practices, due to their roles and responsibilities in the home.

Despite the strengths of the study, some limitations have been identified. This paper intends to apply the socioecological model by considering the individual and community. However, the policy level factors were excluded due to the facts that the policy level factors were not available in the data, which is a deficiency in this study. The survey data is cross sectional, therefore causal inference cannot be made about potential relationships between explanatory variables and combined WASH. The lack of observation of survey data collection means another family member or proxy could have provided survey responses, instead of the mother, and therefore, responses to WASH related survey items may not be consistent with the actual experiences of the mother. Survey data on mothers’ access to improved water sources did not differentiate between mothers from the same village, as almost all households in a village have the same type of water source. This study identified missing survey data such as De Facto mothers who were excluded from participating in the NDHS 2016, and mothers with children over the age of five, which may have affected the study results. Improved WASH was used throughout the paper with reference of guide to DHS statistics-7. However, it lacks using safety managed water source, sanitary toilet, basic services and limited uses in the the World Health Organization/United Nations International Children’s Emergency Fund Joint Monitoring Programme.

## Conclusion

The WASH is significantly associated with the level of education, caste/ethnicity, wealth index, ecology, and distance to water source for mothers. Exposure to newspaper and television were significantly associated with combined WASH. There must be established WASH literacy and the WASH should be promoted as an individual responsibilities and community supports. This means there must be developed better understanding of an individual drivers of WASH uptake. Further research is needed to assess the health effects of combined WASH on children, mothers and other family members. Tangible indicators for scientific measurement of each component of WASH are also needed. This could be through observation of household activity to accurately measure the prevalence of WASH access and practice by mothers. Critical evaluation of individual and community factors would provide understanding of their influence access to and use of WASH services.

The actions of health and beyond the health sectors may be instrumental in improving quality WASH services in Nepalese households. This study found advocacy, WASH services and WASH policy and guidelines with reference to existing knowledge should be effectively launched in the community and combined WASH must be provided to all mothers. The outcome of this study will advocate for the prioritization of WASH programs for mothers with children under five years in Nepal.

## Supporting information

S1 Table Sociodemographic characteristics of respondents. (TIF)

S1 Fig Model fitness test. (TIF)

S2 Fig Socioecological model. (TIF)
