# Supplementary figures and images for: Multi-level factors associated with utilization of water, sanitation and hygiene services by mothers in Nepal
Source: PLoS One. 2024 Mar 20;19(3):e0283379. doi: 10.1371/journal.pone.0283379 (PMC10954160; doi:10.1371/journal.pone.0283379)

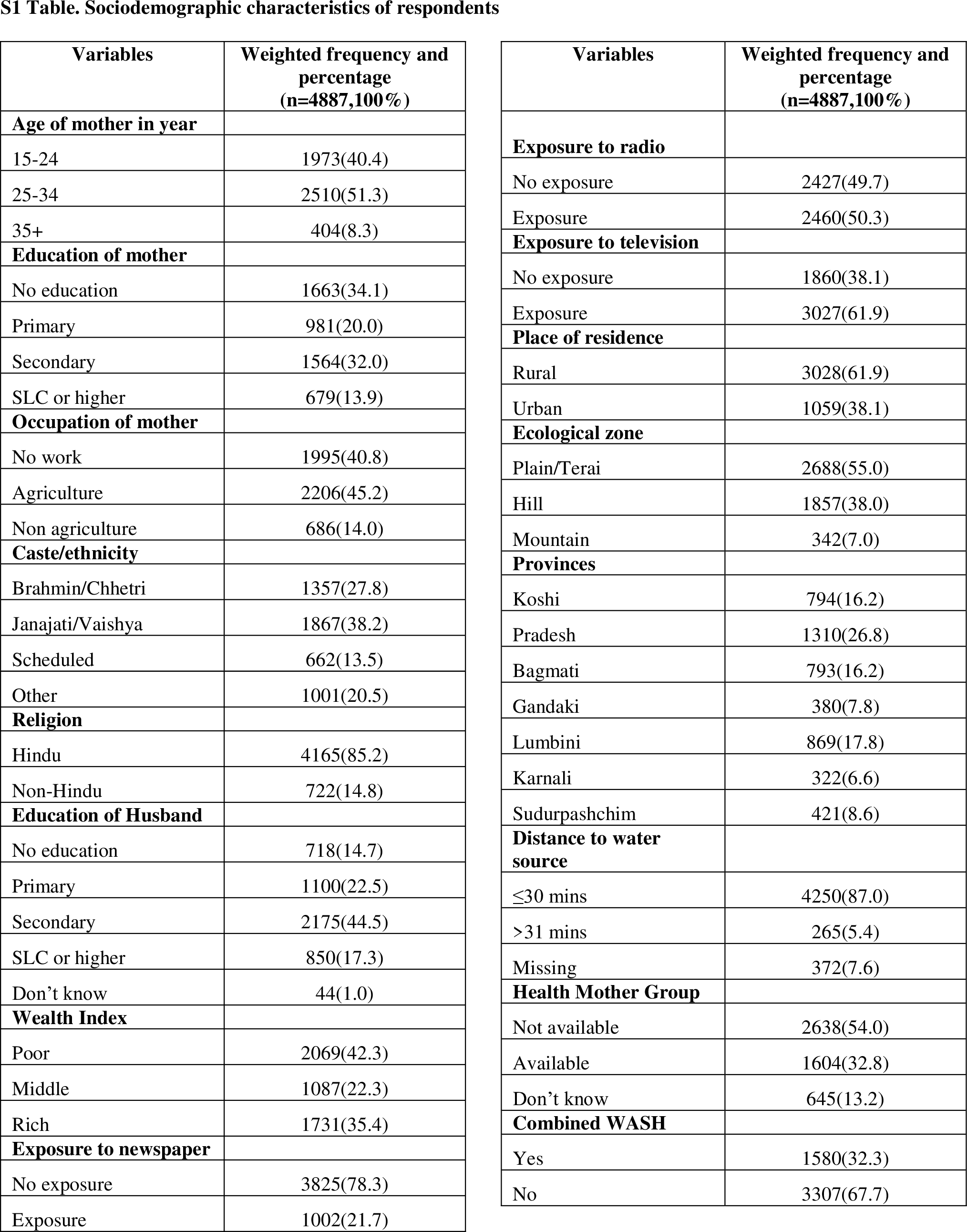

Supplement: S1 Table — (TIF) [file pone.0283379.s001.tif]

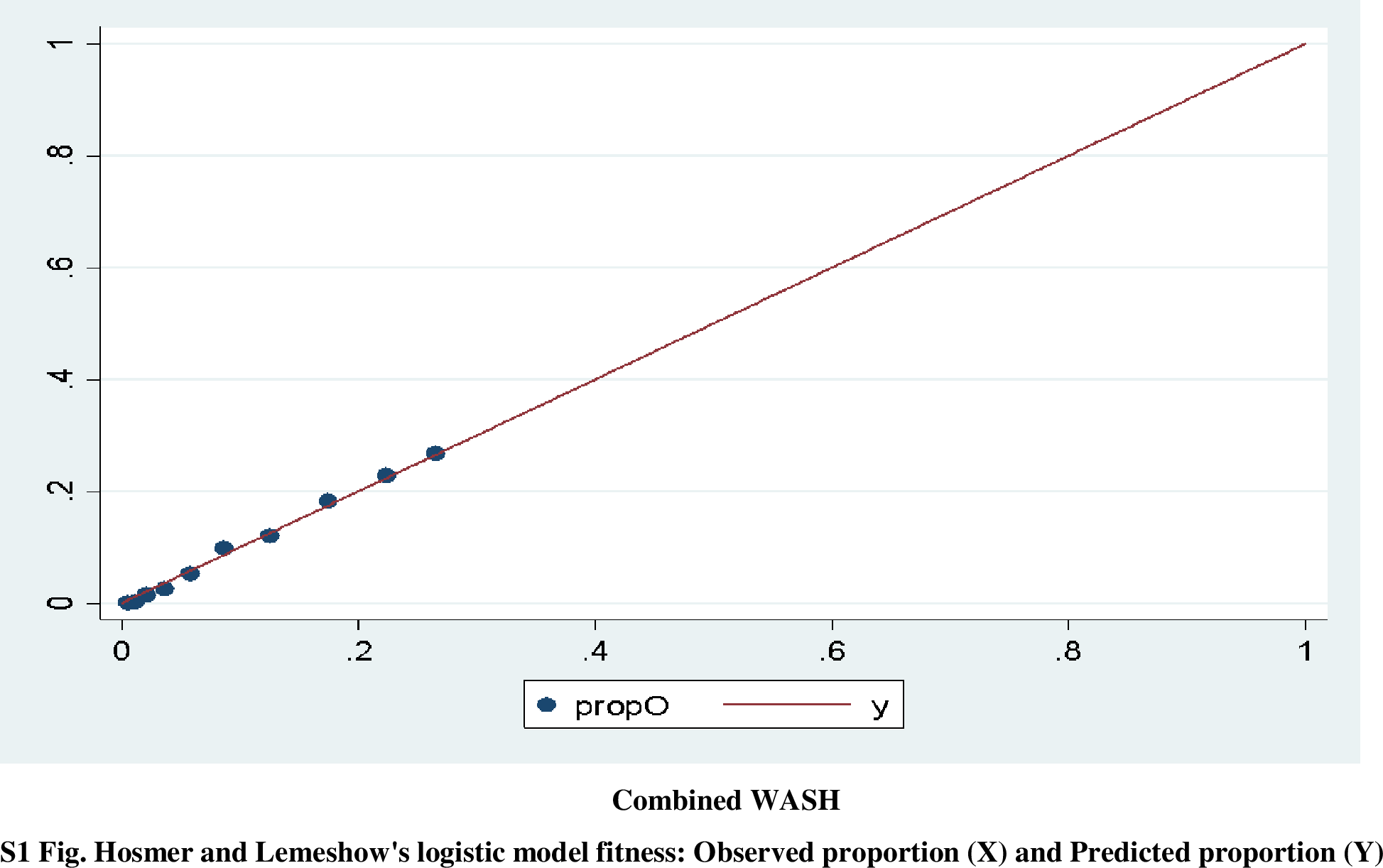

Supplement: S1 Fig — (TIF) [file pone.0283379.s002.tif]

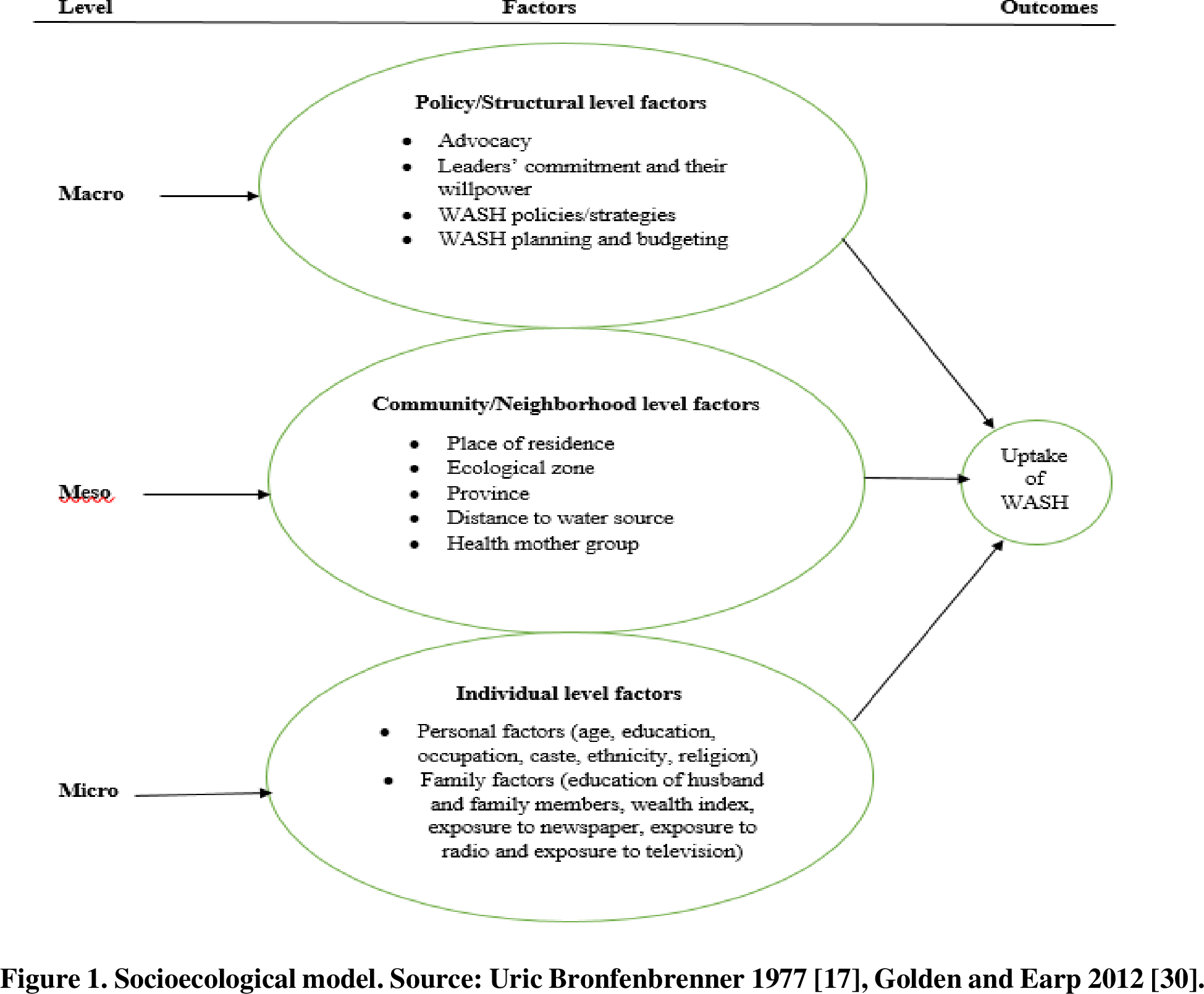

Supplement: S2 Fig — (TIF) [file pone.0283379.s003.tif]
